# Chronic and Binge Alcohol Ingestion Increases Truncated Oxidized Phosphatidylcholines in Mice Lungs Due to Increased Oxidative Stress

**DOI:** 10.3389/fphys.2022.860449

**Published:** 2022-05-24

**Authors:** Corynn N. Appolonia, Kaelin M. Wolf, Charles N. Zawatsky, Resat Cinar

**Affiliations:** Section on Fibrotic Disorders, National Institute on Alcohol Abuse and Alcoholism, National Institutes of Health, Rockville, MD, United States

**Keywords:** alcohol, oxidized phospholipids, lung injury, PGPC, POVPC, IL-17, LPS

## Abstract

Heavy alcohol drinking has negative health effects in multiple organs. It predisposes lungs to inflammatory conditions associated with acute lung injury and increased incidence of pneumonia and sepsis, which may lead to death due to acute respiratory distress syndrome in some individuals with alcohol use disorder (AUD). In general, rodent models of alcohol exposure either do not recapitulate multiple organ injuries as seen in humans or require longer duration to establish tissue injury and inflammation. The recently introduced NIAAA model of alcohol-induced liver injury, characterized by a marked increase in steatosis and liver damage with 10 days of a liquid diet containing 5% ethanol followed by a single ethanol binge (5 g/kg). Therefore, we employed this model to explore the status of surfactant phospholipids, oxidative stress, tissue injury markers and inflammatory cytokines in lungs. In lungs of C57BL/6J mice, the alcohol feeding significantly increased levels of the surfactant phospholipid dipalmitoyl phosphatidylcholine (DPPC) as well as the truncated oxidized phosphatidylcholines palmitoyl oxovaleryl phosphatidyl-choline (POVPC), palmitoyl glutaryl phosphatidyl-choline (PGPC), palmitoyl oxo-nonanoyl phosphatidyl-choline (ALDO-PC), and palmitoyl azelaoyl phosphatidyl-choline (PAzePC) at 9 h post-binge. Additionally, gene expression of the enzymes catalyzing lipid oxidation, such as arachidonate 15-lipoxygenase (Alox15), prostaglandin synthase 2 (Ptgs2), Cytochrome P450 2E1 (Cyp2E1) and NADPH oxidase 1 (Nox1) were significantly increased. Furthermore, ethanol increased levels of the inflammatory cytokine Interleukin-17 in bronchoalveolar lavage fluid. In conclusion, the NIAAA alcohol feeding model might be suitable to study alcohol-induced lung injury and inflammation.

## Introduction

Alcohol use disorder (AUD) is characterized by the inability to stop or control alcohol consumption despite negative consequences. According to the 2019 National Survey on Drug Use and Health, approximately 14 million people ages 18 and older in the United States had AUD, which is 5.6% of this age group (National Survey on Drug Use and Health, 2019a; National Survey on Drug Use and Health, 2019b).

The negative health consequences of AUD are well-established. Historically, liver injury is recognized as the predominant consequence of alcohol consumption in peripheral organs. Aside from the liver, alcohol also damages other organs, including the lungs (Mehta and Guidot, 2017), which is the major focus of this study.

Possible mechanisms for alcohol-induced lung injury include increased oxidative stress, altered tissue remodeling (Guidot and Roman, 2002a), and dysregulated lung inflammation (Massey et al., 2015a). Eventually, these can result in a defective immune response during host defense immunity. All of these mechanisms make the lungs susceptible to inflammatory and progressive lung disorders (Guidot and Roman, 2002b). While alcohol primes the lungs for secondary insult resulting in various pathological conditions, we need to understand common pathological pathways triggered by alcohol in appropriate experimental models, which will aid in the identification of therapeutic targets for alcoholic lung injury. Recently, the NIAAA alcohol drinking model (chronic and single binge alcohol) has been advanced as a rodent model of alcohol-induced liver injury with marked increases in steatosis and tissue injury markers like ALT and AST (Bertola et al., 2013). Additionally, the same model was also shown to promote lung inflammation and hyperresponsiveness to broncho-constrictive stimuli after alcohol binge (Poole et al., 2019). However, the cellular mechanisms of lung injury observed in this model have not been explored in detail. Therefore, we employed the NIAAA alcohol drinking model in C57Bl/6J mice to explore the status of surfactant phospholipids, oxidative stress markers and inflammatory cytokines and chemokines, which are critical indicators of lung health status.

We found the NIAAA alcohol feeding model reasonably well replicates the manifestations of lung inflammation and injury such as dysregulated surfactant phospholipids, increased oxidative stress, and elevated truncated oxidized phospholipids and inflammatory cytokines.

## Methods

### Animals and Ethanol Feeding

**FIGURE 1:**
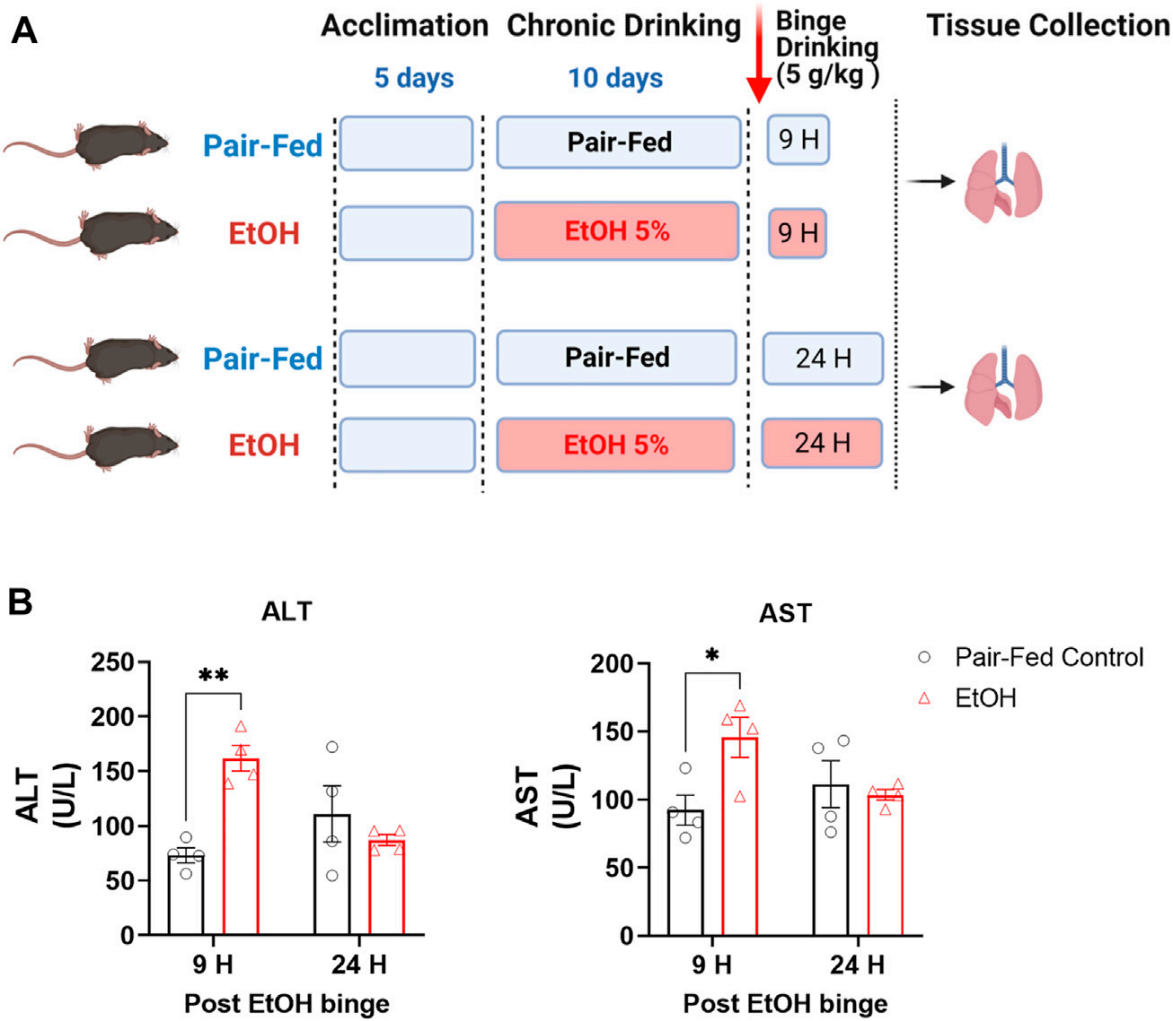
Experimental paradigm of the NIAAA alcohol feeding model in mice. **(A)** Schematic presentation of the NIAAA alcohol feeding model. **(B)** Levels of alanine transaminase (ALT) and aspartate transaminase (AST) in serum. Data represent mean +S.E.M from 4 mice per group. Data were analyzed by one-way ANOVA followed by Dunnett’s multiple comparisons test. * (*p* < 0.05), ** (*p* < 0.01) indicates significant difference from the pair-fed control group.

All animal procedures were conducted in accordance with the rules and regulations of the Institutional Animal Care and Use Committee of the National Institute on Alcohol Abuse and Alcoholism (NIAAA) under the protocol LPS-GK1. Twelve-week-old male C57BL/6J mice were obtained from The Jackson Laboratory (Bar Harbor, ME, United States). Mice were exposed to either control or ethanol-containing Lieber-DeCarli liquid diet. The NIAAA model of chronic and binge ethanol feeding was followed as described (Bertola et al., 2013). In brief, mice were fed a control liquid diet for 5 days, after which they divided into two groups: a pair-fed control group and an ethanol-fed group. For the next 10 days, the ethanol-fed group received a liquid diet containing 5% ethanol, whereas the pair-fed control group received an isocaloric control liquid diet. On day 11, the pair-fed control mice received maltose dextrin *via* gavage, and the ethanol-fed mice received 5 g/kg ethanol by gavage. The experimental paradigm is depicted in Figure 1A. Four- five mice used per group.

Following the gavage, mice were sacrificed by decapitation. Serum was obtained from by retroorbital bleeding. Left lung tissue was snap-frozen in liquid nitrogen for protein and mass spectrometry analysis. Portions of right lung tissue were placed in RNAlater (ThermoFisher Scientific) and were kept at 4°C overnight for RNA extraction.

### ALT and AST Measurements

Serum levels of alanine transaminase (ALT) and aspartate transaminase (AST) were measured using EnzyChrom™ Alanine Transaminase Assay Kit and EnzyChrom™ Aspartate Transaminase Assay Kit, respectively (BioAssay Systems).

### Real-Time Quantitative PCR Analysis

RNA extraction was performed using RNeasy Mini Kits from Qiagen (Valencia, CA). One microgram of total RNA was reverse transcribed to cDNA using iScript cDNA synthesis kit (Bio-Rad, Hercules, CA). Expression of the target genes was quantified using gene-specific primers and PowerSYBRGreen master mix (ThermoFisher Scientific) in a QuantStudio 3 Real-Time PCR instrument (ThermoFisher Scientific). Predesigned mouse Alox15 (QT00111034), Ptgs1 (QT00155330), Ptgs2 (QT00165347), Nox1 (QT00140091), Nox4 (QT00126042), Aldh2 (QT00158368), Adh1 (QT00093520), Cyp2e1 (QT00112539), Cybb (QT00139797) primers were purchased from Qiagen (Valencia, CA). Fold changes in gene expression relative to the house-keeping gene TATA-Box Binding Protein (Tbp, QT00198443) were determined.

### Mass Spectrometry Measurements

Levels of phosphatidylcholines (DPPC, PAPC, POPC) and truncated oxidized phosphatidylcholines (PGPC, POVPC, PAzePC, 16-9-ALDOPC) were measured in lungs by liquid chromatography/tandem mass spectrometry (LC-MS/MS). Left lung tissue was homogenized in 0.5 ml of ice-cold methanol/Tris buffer (50 mM, pH 8.0), 1:1, containing 250 ng of [^2^H_4_] Platelet Activating Factor C-16 ([^2^H_4_] PAF C-16) as internal standard. Homogenates were extracted three times with CHCl_3_: MeOH (2:1, vol/vol), dried under nitrogen flow and reconstituted with MeOH after precipitating proteins with ice-cold acetone. LC-MS/MS analyses were conducted on an Agilent 6,470 triple quadrupole mass spectrometer (Agilent Technologies) coupled to an Agilent 1200 LC system. Analytes were separated using an Agilent InfinityLab Poroshell 120 EC-C18 column (3.0 × 100 mm, 2.7 micron). Gradient elution mobile phases consisted of 0.1% formic acid in H_2_O (phase A) and 0.1% formic acid in MeOH (phase B). Gradient elution (350 μL/min) was initiated at 10% B followed by a linear increase to 50% B at 0.5 min and then another linear increase to 85% B at 3 min and maintained until 14 min before increasing linearly to 100% B by 18 min and maintained until 38 min. The mass spectrometer was set for Agilent Jet Stream high sensitivity electrospray ionization operated in positive ion mode. The source parameters were as follows: capillary voltage, 3500 V; gas temperature, 300°C; drying gas flow, 5 L/min; sheath gas temperature, 250°C; sheath gas flow, 10 L/min. Nitrogen was used as the nebulizing gas. Collision-induced dissociation was performed using nitrogen. Levels of each compound were analyzed by multiple reaction monitoring. The molecular ion and fragment for each compound were measured as follows: m/z 734.6→183.9 for DPPC, m/z 782.6→183.9 for PAPC, m/z 746.6→183.9 for POPC, m/z 610.4→183.9 for PGPC, m/z 594.4→183.9 for POVPC, m/z 650.4→183.9 for 16-9-ALDOPC, m/z: 482.4 →104.1 for Lyso-PAF C16:0, m/z:524.4 →183.9 for PAF C16:0 m/z 666.4→183.9 for PAzePC, m/z 528.4→184 for [^2^H_4_]PAF C16:0. Analytes were quantified using MassHunter Workstation LC/QQQ Acquisition and MassHunter Workstation Quantitative Analysis software (Agilent Technologies). Levels of lipids in the samples were measured against standard curves.

### LPS-Induced Acute Lung Injury

To induce acute lung injury, 16-week-old mice received a single oropharyngeal instillation of lipopolysaccharide (LPS) (50 µg/mouse) as described previously (Park et al., 2020). LPS (L4391, Millipore Sigma) was dissolved in sterile saline and administered at 50 µg/mouse in 40 µL after anesthesia induced by ketamine/xylazine. In this part of the study both pair-fed control and ethanol-fed mice received sterile saline. The mice were studied 24 h after LPS or saline instillation, as depicted schematically in Figure 4A.

### BALF Collection From Mice

BALF was collected from anesthetized mice by lavaging lungs three times with 0.8 ml Hanks’ balanced salt solution without calcium and magnesium (HBSS) (Sigma Aldrich). Supernatants were collected as BALF for further analyses.

### Lactate Dehydrogenase Cytotoxicity Assay

CyQuant LDH cytotoxicity assay kit (Invitrogen) was used to assess LDH activity in BALF samples, as detailed in the product manual.

### Level of Albumin in BALF

Albumin levels were measured by Mouse Albumin Elisa Assay kit obtained from Bethyl Laboratories. BALF samples from pair-fed control and ethanol-fed mice were diluted at 1:1000 ratio, whereas samples from LPS group were diluted at 1:2000 ratio.

### Levels of Total Protein in BALF

Total protein in BALF samples was measured using Pierce BCA protein assay kit as detailed in the product manual (ThermoFisher Scientific).

### Cytokine Measurements

Levels of monocyte chemoattractant protein-1 (MCP-1), tumor necrosis factor alpha (TNFα), interleukin 1 beta (IL-1β) and Interleukin 17 (IL17) in mouse BALF were measured using the mouse CCL2 quantikine ELISA Kit, (R&D systems, MJE00B), mouse TNF-alpha quantikine ELISA Kit, (R&D systems, MTA00B), mouse IL-1β quantikine ELISA Kit, (R&D systems, MLB00C), mouse IL-17 quantikine ELISA Kit, (R&D systems, M1700), respectively.

### Statistical Analysis

Statistical analysis was performed using GraphPad Prism 9 (GraphPad Software Inc.). Two-way ANOVA followed by Sidak’s multiple comparisons test was performed. *p* < 0.05 was considered significant.

## Results

### Oxidative Stress in Lungs Increased by Chronic and Single Binge Ethanol Feeding

The NIAAA mouse model of chronic with binge ethanol feeding was utilized in this study because the model is known to better induce liver injury, inflammation and fatty liver, as compared to ethanol feeding models without addition of a binge or secondary insult. The chronic with binge model is also thought to better mimic the drinking patterns of people with an AUD (Bertola et al., 2013). To verify that the ethanol feeding model induced liver injury, we measured serum alanine transaminase (ALT) and aspartate transaminase (AST) levels in pair-fed (PF) control mice and ethanol-fed (EtOH) mice. ALT and AST were significantly elevated in the serum of EtOH mice as compared to PF mice at the 9-h time point only (Figure 1B), indicating significant acute liver injury, which is then mostly resolved by 24-h post-Ethanol binge.

**FIGURE 2:**
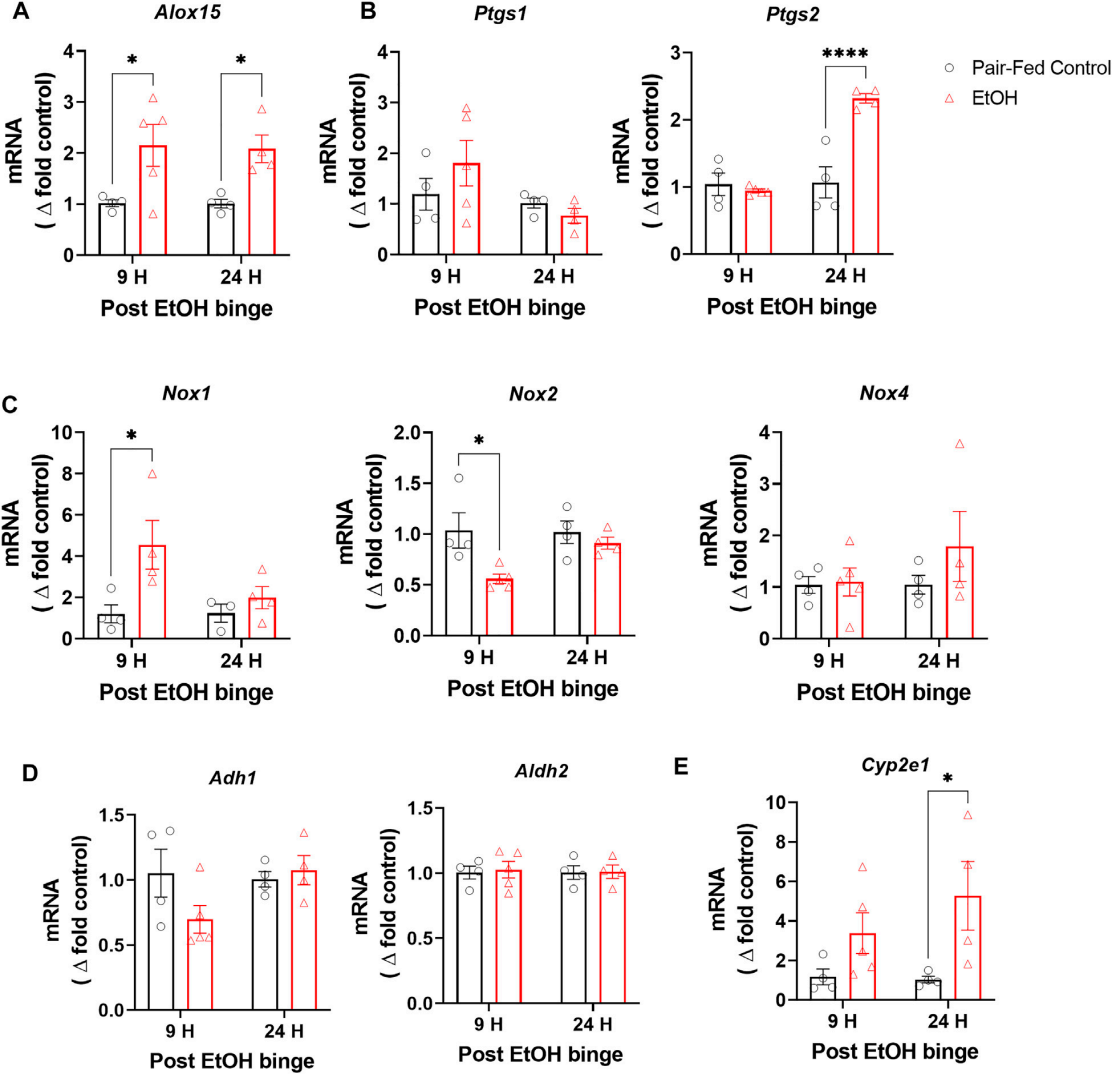
Chronic and single binge ethanol feeding increased expression of oxidative enzymes in mouse lungs. Gene expression of oxidative enzymes arachidonate 15-lipoxygenase (Alox15) **(A)**, Prostaglandin synthase 1 and 2 (Ptgs1 and Ptgs2) **(B)**, NADPH oxidase 1, 2 and 4 (Nox1, Nox2 and Nox4) **(C)**, Alcohol dehydrogenase 1 (Adh1) and Alcohol dehydrogenase 2 (Aldh2) **(D)**, Cytochrome P450 2E1 (Cyp2E1) **(E)** in lungs either pair-fed or ethanol fed mice. Data represent mean +S.E.M from 4-5 mice per group. Data were analyzed by one-way ANOVA followed by Dunnett’s multiple comparisons test. * (*p* < 0.05), **** (*p* < 0.0001) indicates significant difference from the pair-fed control group.

Increased oxidative stress has been postulated as a possible mechanism of alcohol-induced lung injury (Guidot and Roman, 2002a; Massey et al., 2015a). We therefore measured gene expression of oxidative enzymes known to be involved in oxidative stress, such as the inflammatory oxidative enzymes arachidonate 15-lipoxygenase (Alox15), prostaglandin synthase 1 and 2 (Ptgs1 and Ptgs2), and NADPH oxidase 1, 2 and 4 (Nox1, Nox2 and Nox4). Alox15 gene expression was significantly increased 9 h after the binge and then remained elevated for 24 h (Figure 2A). Ptgs2 gene expression was only increased at 24 h after the binge whereas no change was observed in Ptgs1 expression (Figure 2B). Nox1 expression was significantly increased and Nox2 was decreased at 9 h after the binge, which then normalized at 24 h post-binge, whereas we have not observed any changes in Nox4 expression in lung post-binge (Figure 2C). Multiple alcohol metabolizing enzymes such as alcohol dehydrogenase 1 (Adh1), alcohol dehydrogenase 2 (Aldh2) and Cytochrome P450 2E1 (Cyp2E1) are functionally expressed in lungs (Oyama et al., 2005; Mouton et al., 2016). They may also contribute oxidative stress in lungs in addition to alcohol metabolism (Kaphalia et al., 2014). We found that alcohol has not changed gene expression levels of Adh1 and Aldh2 (Figure 2D) whereas expression level of Cyp2e1 is significantly increased (Figure 2E). These results indicate increased oxidative stress in lungs following chronic ethanol and single binge. Since we found significant and sustained elevation in gene expression of several oxidative enzymes, we further explored its functional consequences on lung phospholipid homeostasis.

### Chronic Ethanol and Single Binge Dysregulate Surfactant Phospholipid Homeostasis and Increases Truncated Oxidized Phosphatidylcholines

**FIGURE 3:**
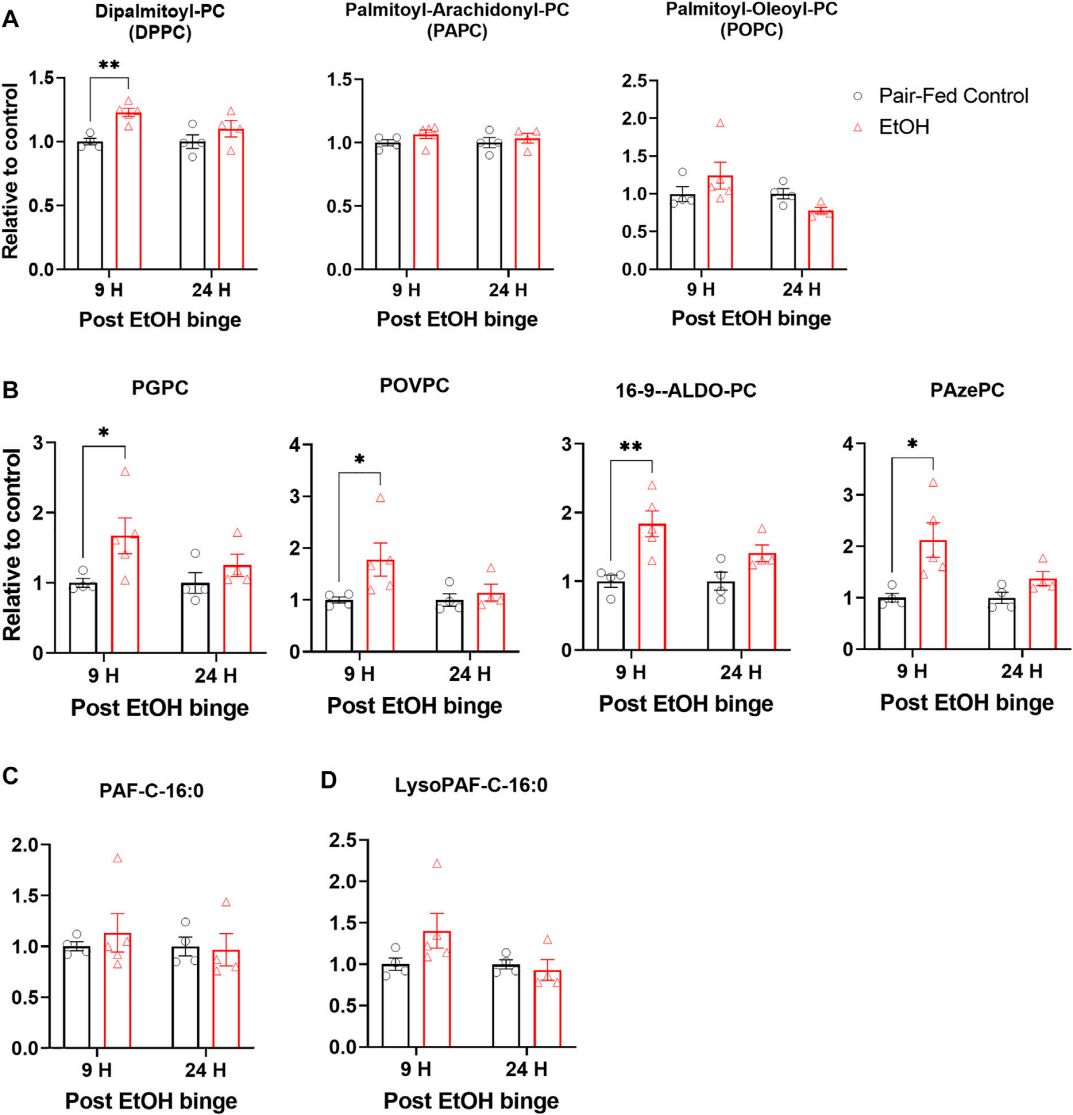
Chronic and single binge ethanol feeding induced elevation of truncated oxidized phospholipids in muse lungs. **(A)** Levels of surfactant phospholipid dipalmitoyl phosphatidyl choline (DPPC), and cellular phospholipids palmitoyl-arachidonoyl phosphatidyl choline (PAPC), palmitoyl-oleoyl phosphatidyl choline (POPC) in lungs. **(B)** Levels of truncated oxidized phosphatidyl cholines, Palmitoyl Glutaryl Phosphatidyl Choline (PGPC), Palmitoyl Oxovaleryl Phosphatidyl Choline (POVPC), Palmitoyl Oxo-nonanoyl Phosphatidyl Choline (ALDO-PC), and Palmitoyl Azelaoyl Phosphatidyl Choline (PAzePC). Platelet activating factor C16:0 (PAF C16:0) **(C)**. Lyso-PAF C16:0 **(D)**. Data represent mean +S.E.M from 4-5 mice per group. Data were analyzed by one-way ANOVA followed by Dunnett’s multiple comparisons test. * (*p* < 0.05), ** (*p* < 0.01) indicates significant difference from the pair-fed control group.

Surfactant phospholipid homeostasis is critical for lung function and host defense. Surfactant phospholipids predominantly consist of dipalmitoyl phosphatidylcholine (DPPC) that about 60% of surfactant lipids in the lungs consist of DPPC. Besides, cellular phospholipids with mono or poly unsaturated fatty acid moieties such as palmitoyl arachidonoyl phosphatidylcholine (PAPC), palmitoyl oleoyl phosphatidylcholine (POPC), and palmitoyl linoleoyl phosphatidylcholine (PLPC) could also involve multiple inflammatory and oxidative processes. Previously, it was shown that chronic alcohol feeding for 6–8 weeks increased levels of phosphatidylcholines (Liau et al., 1981). Using the NIAAA model, we found that DPPC was significantly increased 9 h after the single binge (Figure 3A). Although there is a slight trend towards increased levels of PAPC and POPC, these changes are not statistically significant (Figure 3A).

One of the consequences of an ethanol-induced increase in oxidative stress and in the expression of Alox15 in lungs would be lipid oxidation and generation of truncated oxidized phospholipids (tOxPLs). tOxPLs are bioactive and can have harmful effects on tissues (Karki and Birukov, 2020). For example, the tOxPLs of PAPC (lyso-PC, POVPC, and PGPC) increased endothelial cell permeability in a dose-dependent manner and were barrier disruptive (Birukova et al., 2013). Importantly, tOxPLs could contribute to lung injury, and they are lipid mediators of acute respiratory distress syndrome in lungs (Ke et al., 2019). To explore the status of tOxPLs in lungs in the NIAAA model, we measured the levels of tOxPLs such as palmitoyl oxovaleryl phosphatidyl choline (POVPC), palmitoyl glutaryl phosphatidyl choline (PGPC), palmitoyl oxo-nonanoyl phosphatidyl choline (ALDO-PC), and palmitoyl azelaoyl phosphatidyl choline (PAzePC). POVPC and PGPC are the tOxPLs of surfactant phospholipid PAPC, while ALDO-PC and PAzePC are the tOxPLs of surfactant phospholipid POPC and PLPC. At the 9-h time point, ethanol significantly increased the levels of POVPC, PGPC, ALDO-PC, and PAzePC in the lungs as compared to control lungs (Figure 3B). At the 24-h time point, mild but not significant increases in the levels of all four tOxPLs were observed (Figure 3B). Truncated oxidized phospholipids are structural analogue of Platelet activator factors (PAFs) and Lyso-PAFs. Indeed, there are evidence that they bind to PAF receptors. Therefore, we also check the levels of PAFs. Alcohol ingestion has not changed the levels of PAF C16:0 (Figure 3C) or lyso-PAF C16:0 (Figure 3D). These results suggest that oxidative stress transiently increases tOxPLs, which act as inflammatory lipid mediators in the lungs of ethanol-fed mice.

### Effects of EtOH Versus LPS on Vascular Permeability, Epithelial and Cell Membrane Integrity, and Cytotoxicity the in Lungs

**FIGURE 4:**
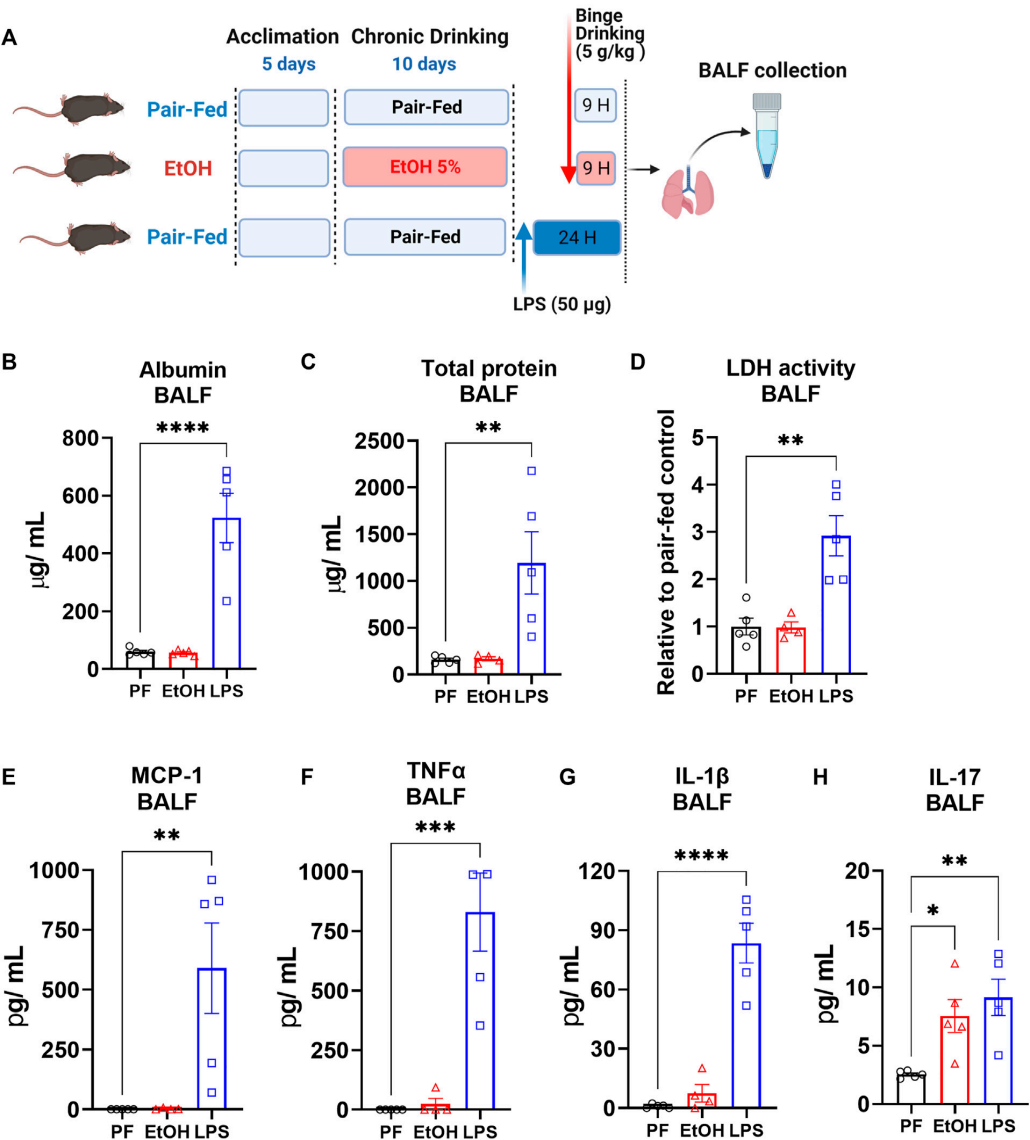
The NIAAA ethanol feeding model did not alter vascular permeability, and epithelial cell membrane integrity but did increase inflammatory cytokines in BALF **(A)** Schematic presentation of ethanol feeding and oropharyngeal LPS (50 ug/mice) induced lung injury model. Levels of **(B)** Albumin, **(C)** Total protein, **(D)** Lactate dehydrogenase (LDH) activity **(E)** MCP-1, **(F)** TNFα, **(G)** IL-1β and **(H)** IL-17 in bronchoalveolar lavage fluid (BALF) from mice 9 h after either pair-fed or ethanol binge following 10 days chronic feeding or 24 h after oropharyngeal LPS instillation. Data represent mean +S.E.M from 4-5 mice per group. Data were analyzed by one-way ANOVA followed by Dunnett’s multiple comparisons test. * (*p* < 0.05), ** (*p* < 0.01), *** (*p* < 0.001), **** (*p* < 0.0001) indicates significant difference from the pair-fed control group.

Hallmarks of acute lung injury include increased vascular permeability, cytotoxicity, and disrupted epithelial and cell membrane integrity (Matute-Bello et al., 2008). It is known that heavy alcohol drinking in individuals with AUD increases vascular permeability and dysregulates epithelial integrity in lungs (Joshi and Guidot, 2007; Burnham et al., 2009). To explore the status of acute lung injury in the NIAAA model, we collected bronchoalveolar lavage fluid (BALF) from mice 9 h after the ethanol binge feeding and measured albumin, lactate dehydrogenase (LDH) and total protein in BALF as indicators of increased vascular permeability, cytotoxicity and disrupted epithelial integrity. For comparison, we made similar measurements in the BALF of mice following oropharyngeal LPS instillation, an established model of acute lung injury. Levels of albumin, total protein and LDH were not altered in BALF by 10 days chronic ethanol followed by a single binge (Figure 4), whereas LPS significantly increased albumin, total protein and LDH activity in BALF (Figures 4A–C), indicating acute lung injury.

### Chronic Ethanol Plus Single Binge Feeding Increased IL-17 Protein Level in BALF

To explore status of inflammatory cytokines in lung after either chronic feeding plus single ethanol binge or LPS instillation, we measured inflammatory cytokines in BALF. LPS instillation significantly increased the BALF content of monocyte chemoattractant protein-1 (MCP-1), tumor necrosis factor alpha (TNFα), interleukin 1 beta (IL-1β) and Interleukin 17 (IL17) (Figures 4B–D). in contrast, ethanol did not significantly alter the BALF levels of MCP-1, TNFα and IL-1β (Figures 4E–G) but significantly increased IL17 to levels seen after LPS instillation (Figure 4H).

## Discussion

We have demonstrated that chronic ethanol followed by a single binge increases gene expression of oxidative stress-related enzymes, levels of truncated oxidized phospholipids as a consequence of oxidative stress and inflammatory cytokine IL-17 in lungs of C57BL/6J mice using the NIAAA alcohol model. Several studies have investigated the role of oxidative stress in alcohol-induced lung injury (Moss et al., 2000; Guidot and Roman, 2002b; Velasquez et al., 2002; Polikandriotis et al., 2006; Jensen et al., 2013). In a clinical study, heavy alcohol use was associated with a four-fold decrease in glutathione (GSH) levels in the BALF, regardless of smoking status (Moss et al., 2000; Yeh et al., 2007). It is known that alcohol can promote lung injury by increasing oxidative stress in heavy alcohol drinkers (Moss et al., 2000). Previously, long-duration of alcohol exposure models demonstrated increased oxidative stress in lungs (Holguin et al., 1998). Here, we demonstrated using 10 days of chronic ethanol (5%) and single ethanol binge (5 g/kg) feeding causes an increased expression of oxidative enzymes (Alox15, Ptgs2, Nox1,Cyp2E1), which may contribute to oxidative stress and lung inflammation *via* lipid oxidation with generating inflammatory bioactive lipids such as truncated oxidized phospholipids (Massey et al., 2015a). Additionally, the NIAAA model caused dysregulation of surfactant phospholipids by increasing their amount in mice, which is in agreement with previously reported data in lungs of 7 weeks ethanol-fed rats (Liau et al., 1981). One of the critical outcomes of increased oxidative stress in lungs could be generation of truncated oxidized phospholipids such as POVPC, PGPC, ALDO-PC, and PAzePC. These tOxPLs are proinflammatory through binding to diverse receptors including Platelet-activating factor receptor and Toll-like receptor 4, among others (Bochkov et al., 2010). tOxPLs are also lipid inflammatory mediators of acute respiratory distress syndrome (ARDS) (Ke et al., 2019). tOxPLs are formed by an enzymatic mechanism *via* direct action of 15-lipoxygenase (15-LO) on phospholipids, and/or indirect formation through eicosanoid incorporation into lyso-phospholipids. Increased gene expression of ALOX15, encodes 15-LO, ptgs2, encodes cyclooxygenase-2, and Cyp2e1, encodes Cytochrome P450 2E1. Although we have not measured direct functional activity and protein levels of these enyzmes, one of the critical outcomes of the increased oxidative stress would be an increased generation of tOxPL. Indeed, we found that ethanol ingestion increased truncated oxidized phospholipids such as POVPC, PGPC, ALDO-PC, and PAzePC as a result of increased oxidative stress in lungs.

Acute binge drinking in healthy individuals increases serum levels of LPS and inflammatory cytokines (Bala et al., 2014). Additionally, the increase in serum endotoxin levels in chronic drinkers has been proposed as a critical factor for alcohol-induced tissue injuries (Wang et al., 2010). Chronic ethanol exposure was also shown to potentiate LPS-induced lung injury in mice (Massey et al., 2015b). Here, we showed that chronic ethanol followed by a single binge increased IL-17 levels in BALF similarly as seen in LPS induced lung injury (Figure 4H). However, the inflammatory cytokines MCP-1, TNFα and IL-1β were only significantly elevated in BALF by LPS induction. A slight IL-1β increase together with significant IL-17 elevation by ethanol feeding suggests an existing inflammatory microenvironment in the lungs, which is not sufficient to induce severe lung injury alone as observed with LPS (Figure 4). Chronic ethanol exposure in mice increased IL-17 related inflammatory responses systemically (Frank et al., 2020). Besides, circulating IL-17 levels are positively correlated with heavy alcohol drinking, and are further increased in patients with alcoholic liver disease (Xu et al., 2020). Furthermore, alcohol-induced overactivation of IL-17 signaling contributes to liver injury and fibrosis, which could be prevented by attenuating IL-17 signaling (Xu et al., 2020). IL-17 is primarily secreted by neutrophils and lymphocytes. It was shown that the NIAAA model increased neutrophil and lymphocyte infiltration in lungs (Poole et al., 2019). Therefore, infiltrated neutrophils and/or lymphocytes might be responsible for increased IL-17 levels in BALF. Recent study demonstrated using 8 weeks alcohol drinking model in mice that alcohol-induced IL-17 and Th17 immune response promotes lung fibroblast differentiation to myofibroblasts (Neveu et al., 2019), eventually this could cause fibroproliferative microenvironment in lungs of chronic alcohol drinkers. Therefore, IL-17 might be an important mediator in alcohol-induced lung injury and subsequent fibroproliferative disorders, which warrants further investigation.

We tested whether 10 days chronic ethanol followed by a single binge feeding could be sufficient to induce acute lung injury. As a basis for comparison, we also used the LPS-induced acute lung injury model, which causes severe lung injury. We found that ethanol by itself as used in the NIAAA model was not sufficient to induce severe acute lung injury comparable to that seen in the LPS-induced lung injury model (Figure 4). Although, we could not see a severe lung injury by Alcohol compared to the LPS, this suggest there might be delayed chronic injury by alcohol since it increased circulating LPS levels in heavy drinkers. Besides, It is known that alcohol has a priming effect in sensitizing the lungs to secondary insults such as lipopolysaccharide (Massey et al., 2015b) or bleomycin (Sueblinvong et al., 2014). Dysregulation of surfactant phospholipids and increases in truncated oxidized phospholipids may be part of the mechanism by which alcohol consumption and the resulting oxidative stress increases susceptibility to acute lung injury (Guidot and Roman, 2002a), enhances lipopolysaccharide-induced lung injury (Massey et al., 2015b), and exacerbates bleomycin-induced lung fibrosis (Sueblinvong et al., 2014). Alcohol use disorder was characterized as the only independent risk factor known to increase the chance of any given at-risk individual developing ARDS (Kershaw and Guidot, 2008). ARDS is a severe form of acute lung injury with a mortality rate of about 40% and without effective pharmacological treatment (Kershaw and Guidot, 2008). Specifically, patients who misuse alcohol were 3.7 times more likely to develop ARDS as compared to non-AUD individuals (Moss et al., 2003; Kershaw and Guidot, 2008). Additionally, in patients who developed sepsis, the incidence of ARDS was 52% in patients with a history of AUD versus only 20% for patients without a history of AUD (Moss et al., 1996). Therefore, identifying critical mechanisms driving susceptibility to ARDS in individuals with AUD could have important clinical implications for the development of specific therapies. Current study, increased tOxPLs and IL-17 could indicate an inflammatory process in lungs after chronic and a single binge ethanol feeding. Therefore, further studies are warranted to explore alcohol effects on lung health using the NIAAA drinking model with potentially extending the ethanol feeding duration and/or combination of secondary insults.

Heavy alcohol drinking increases incidence of pneumonia and sepsis, which may lead to death due to ARDS in some individuals (Mehta and Guidot, 2017). While the world is significantly impacted by COVID-19 pandemic due to SARS-COV-2 infection, one of the negative health outcomes of heavy alcohol drinking on lungs would be predisposing individuals with AUD for poor prognosis when suffering from SARS-COV-2 associated pneumonia and ARDS (Bailey et al., 2021). Beside the serious and acute health threat represented by COVID-19, there is also growing concern about potential late complications among survivors of an acute COVID-19 infection, such as pulmonary fibrosis. For instance, in patients with COVID who were discharged from hospitals, impaired lung physiology was seen in up to 56% of patients at 6 months and persistent radiological changes were detected in 24% of patients at 12 months (Huang et al., 2021; Wu et al., 2021). tOxPLs are known to activate macrophages to acquire fibroproliferative phenotype (Romero et al., 2015). In the current study, we demonstrated that alcohol ingestion increased tOxPLs in lungs. Since alcohol misuse could dysregulate tissue remodeling in lungs toward fibroproliferative changes and prime lungs for a secondary injury (Guidot and Roman, 2002b; Osna et al., 2021), COVID-19 survivors with AUD may have higher incidence of developing late respiratory complications. With the emerging health crisis caused by COVID-19, it is important to find out whether and how certain co-morbidities, such as AUD, may worsen the prognosis of this potentially fatal viral infection and its long-term sequelae in the lungs. Therefore, future studies combining experimental models of alcohol ingestion and lung injury and/or infection could be instrumental for understanding the effects of alcohol in inflammatory and infectious lung diseases.

## Data Availability

The raw data supporting the conclusions of this article will be made available by the authors, without undue reservation.
